# Whole-genome analysis and antimicrobial resistance phenotype of *Vagococcus fluvialis* isolated from wild *Niviventer*

**DOI:** 10.3389/fmicb.2025.1546744

**Published:** 2025-04-16

**Authors:** Jian Zhou, Ying Liu, Tao Gu, Jingzhu Zhou, Fengming Chen, Yong Hu, Shijun Li

**Affiliations:** ^1^The Key Laboratory of Environmental Pollution Monitoring and Disease Control, School of Public Health, Ministry of Education, Guizhou Medical University, Guiyang, China; ^2^Key Laboratory of Microbio and Infectious Disease Prevention and Control in Guizhou Province, Guizhou Center for Disease Control and Prevention, Guiyang, China

**Keywords:** *Vagococcus fluvialis*, *Niviventer*, whole genome sequencing, genes annotation, drug resistance

## Abstract

*Vagococcus fluvialis* (*V. fluvialis*), a Gram-positive bacterium belonging to the *Enterococcaceae* family, has been associated with human infections, including bacteremia and endocarditis. Its zoonotic potential raises concerns for public health, yet research on its antimicrobial resistance and pathogenicity is still limited. This study aimed to isolate and characterize *V. fluvialis* from wild *Niviventer*, analyze its genomic features (including antimicrobial resistance and virulence genes), and evaluate its antibiotic susceptibility profile to assess potential public health risks. We first isolated *V. fluvialis* (strain 25C42) from the rectum of wild *Niviventer*, confirmed through Matrix-Assisted Laser Desorption/Ionization Time-of-Flight Mass Spectrometry (MALDI-TOF MS) and 16S rRNA gene sequencing. Whole-genome sequencing (WGS) was performed using second-and third-generation technologies, with subsequent quality control and assembly. Six databases including KEGG, COG, CARD and VFDB were used for genome annotation. Antibiotic susceptibility was evaluated according to Clinical and Laboratory Standards Institute (CLSI) guidelines, determining the minimum inhibitory concentrations (MIC) for 16 antibiotics. Strain 25C42 was identified as *V. fluvialis*, confirmed by MALDI-TOF MS and 16S rRNA sequencing. WGS revealed a genome length of 2,720,341 bp, GC content of 32.57%. Functional genomic analysis identified 2,268 genes in the COG database and 2,023 genes in KEGG, highlighting key metabolic and cellular processes. Notably, 119 virulence genes and 65 antimicrobial resistance genes were found, indicating significant resistance potential. Phylogenetic analysis demonstrated a close relationship with other *Vagococcus* species, particularly *V. fluvialis* (ANI 98.57%, DDH 88.6%). Antibiotic susceptibility tests indicated strain 25C42 was resistant to clindamycin, tetracycline, rifampicin, cefoxitin and levofloxacin. Our findings reveal that the wild rodent-derived *V. fluvialis* strain 25C42 harbors clinically relevant antimicrobial resistance determinants and virulence-associated genes. The high genomic integrity and extensive functional gene annotation underscore its metabolic versatility. Notably, strain 25C42 exhibits significant antimicrobial resistance, necessitating ongoing surveillance and research to understand its implications for public health and environmental monitoring, as well as strategies for effective therapeutic intervention.

## 1 Introduction

*Vagococcus fluvialis* (*V. fluvialis*) is a Gram-positive bacterium classified within the genus *Vagococcus* and the family *Enterococcaceae*, closely related to other *enterococci*, such as *Enterococcus*. Originally isolated from chicken manure and river water, *V. fluvialis* was first characterized by Hashimoto et al. (Collins et al., 1989; Schleifer et al., 1985). The specific host species of *V. fluvialis* in animals remains uncertain, however, it was identified in bats by Qin et al. (2021) in China, followed by its detection in bovine urine by Giannattasio-Ferraz et al. (2021) in the United States. Notably, *Niviventer*, a small rodent prevalent in mountainous and forested regions of Asia, particularly in the Yunnan and Guizhou provinces of China, has been recognized as a host for a diverse array of pathogenic bacteria and parasites (Yan, 2021; Tian et al., 2020; Lu et al., 2022).

In recent years, the isolation of *V. fluvialis* from human patients has become increasingly common. Matsuo et al. (2021) reported a case of bacteremia and pressure ulcers associated with *V. fluvialis*, while Jadhav and Pai (2019) documented endocarditis linked to this bacterium. Furthermore, *V. fluvialis* has been isolated from the bile of patients suffering from cholecystitis and from peritoneal fluid in individuals with cirrhosis (Zhang et al., 2023; Kucuk et al., 2022). In China, Ting et al. (2019) isolated *V. fluvialis* from postoperative infected puncture fluid of the lower left femur for the first time. Additionally, this bacterium has been found in human urine on multiple occasions, demonstrating its potential to cause harm to patients (Chen et al., 2024; Kitano et al., 2024).

Researchers have raised concerns regarding the zoonotic transmission of *V. fluvialis* from animals to humans, leading to heightened clinical interest in this organism. Despite an increasing number of reported clinical cases, the direct clinical implications of *V. fluvialis* on human health remain unclear. Moreover, the spectrum of drug resistance and the underlying mechanisms of *V. fluvialis* are yet to be thoroughly investigated. Current studies indicate a potential pathogenicity, however, compared to other common pathogens, research on this bacterium is relatively limited. Other known species within the *Vagococcus* genus exhibit certain pathogenic characteristics and drug resistance profiles (Racero et al., 2021), further accentuating the clinical importance of *V. fluvialis*.

Consequently, it is reasonable to postulate that *V. fluvialis* may pose a risk to human health, however, our understanding of this organism is still inadequate. We report here the first isolation of *V. fluvialis* from the gastrointestinal tract of *Niviventer*. Although previous studies have provided insights into the biological characteristics of this strain (Collins et al., 1989; Schleifer et al., 1985), there is a notable paucity of research focusing on its antimicrobial resistance and pathogenicity, which are critical for clinical diagnosis and treatment. The discovery of *V. fluvialis* in diverse hosts is of paramount significance for elucidating its transmission mechanisms.

To address these gaps, The objectives of this study were: (1) to isolate and identify *V. fluvialis* from wild *Niviventer* using molecular and phenotypic methods; (2) to perform whole-genome sequencing and functional annotation to uncover genomic traits, including antimicrobial resistance and virulence determinants; (3) to determine the antibiotic resistance profile of the isolated strain; and (4) to assess the potential implications of these findings for zoonotic transmission and public health surveillance.

## 2 Methods and materials

### 2.1 Ethics approval

Our study was approved by the Ethics Committee of Guizhou Center for Disease Control and Prevention, approval number: G2019-01. The Ethics Committee agreed that the research was in accordance with the Helsinki Declaration and the Guidelines for the Good Treatment of Animals.

### 2.2 Source of strains

A collection of wild rats were conducted in Jinping County, Qiandongnan City, Guizhou Province. Traps were set overnight and retrieved the following morning. Live mice were euthanized through cervical dislocation under ether anesthesia to ensure a rapid and painless process, this method of euthanasia is recommended in animal euthanasia guidelines in China and the United States [Lu et al., 2021; National Technical Committee for Standardization of Laboratory Animals (SAC/TC 281), 2021]. This research complied with all relevant wildlife protection laws, ensuring the welfare of the captured animals. The species, size, and sex of the specimens were identified according to the Handbook of Important Medical Animals in China (Lu, 1982). After identification, the mice were promptly dissected in the local disease prevention and control center’s laboratory under sterile conditions. Lung tissues (approximately 500 mg) were excised using surgical scissors and immediately inoculated into 1.5 mL of brain heart infusion liquid containing 20% glycerol for preservation. The specimens were homogenized and plated onto Columbia blood agar plates, which were then incubated at 37°C for 48 h to observe microbial growth. Single colonies were isolated for further purification on additional Columbia blood agar plates. The initial identification of the purified colonies was conducted using Matrix-Assisted Laser Desorption/Ionization Time-of-Flight Mass Spectrometry (MALDI-TOF MS), targeting the isolation of potentially significant microbial strains for subsequent identification and analysis. Finally, a strain suspected of *V. fluvialis* (Strain 25C42) was cultured in a rectal specimen of a Niviventer, and the identification score of MALDI-TOF MS was 8.285.

### 2.3 Identification of 16S rRNA gene

Strain 25C42 was isolated and subsequently purified. DNA extraction from the strain was performed using a nucleic acid extraction kit (Hangzhou Baiyi Technology Co., Ltd.). The 16S rRNA gene was amplified via PCR using the primers 27F (5′-AGTTTGATCMTGGCTCAG-3′) and 1492R (5′-AGTTTGATCMTGGCTCAG-3′). Reaction system total 50 ul: Premix Taq 25 ul; Primer 27F 2 ul; Primer 1492R 2 ul; Template DNA 2 ul; Water 19 ul. The reaction condition of PCR amplification was 94°C for 10 min. 94°C 45 s, 52°C 60 s, 72°C 60 s, 40 cycles; 72°C for 10 min. The resultant PCR products were sequenced, and the obtained sequences were subjected to a comparative analysis against the NCBI BLAST database. Homologous sequences with high similarity from GenBank were downloaded for evolutionary analysis.

Phylogenetic reconstruction based on 16S rRNA gene sequences was performed using MEGA 11 software. Four distinct phylogenetic trees were generated through the Clustal_W alignment module, employing the following algorithms: Neighbor-Joining (NJ), Maximum Likelihood (ML), Maximum Parsimony (MP), and Minimum Evolution (ME). Branch node confidence was assessed through 1,000 bootstrap replications, with support values expressed as percentages.

### 2.4 Whole-genome sequencing

A hybrid assembly strategy combining second-generation and third-generation (Oxford Nanopore) sequencing data was employed to leverage the high accuracy of second-generation sequencing and the long-read capabilities of third-generation sequencing, yielding a complete and accurate genome assembly of the strain.

Second-Generation Sequencing was performed using advanced technologies provided by the Beijing Genomics Institute. The process began with DNA fragmentation via enzymatic digestion to generate fragments of 200–500 bp in length suitable for sequencing. Adapter ligation was then performed on both ends of the DNA fragments. The adapters consist of a DNA sequence that includes amplification primers, sequencing primers, and barcode sequences. The barcode allows for distinguishing between different samples and ensuring sequencing accuracy. Following adapter ligation, PCR amplification was carried out to construct the sequencing library. The amplification was performed using primers with dual-barcode sequences, and the products were purified before sequencing. The sequencing was conducted on the BGISEQ platform, which utilizes DNA nanoball (DNB) amplification technology. This method forms DNA nanoballs through rolling circle amplification and generates high-quality raw sequencing data in FASTQ format.

Third-generation sequencing was performed using Oxford Nanopore’s platform, following the proprietary library preparation protocols. DNA fragmentation was carried out using Oxford Nanopore’s DNA fragmentation method, and adapters were ligated to the DNA fragments. Library quality control was conducted using the Agilent Bioanalyzer to assess fragment size distribution, ensuring the library was suitable for sequencing. Sequencing was performed on the Oxford Nanopore platform using electrophoretic migration to load DNA samples into nanopore chips for real-time single-molecule sequencing. Data acquisition and preliminary processing were carried out using the Oxford Nanopore MinKNOW software, and raw sequencing data were obtained in FASTQ format.

The hybrid assembly strategy combined both second-and third-generation sequencing data using the Micro IBS Analyzer software (Beijing MicroFuture Technology Co., Ltd.) for pathogen genome assembly and identification. First, second-generation sequencing data underwent quality control using Trimmomatic (v0.39) to remove low-quality reads. Third-generation sequencing data were filtered and corrected using the PBJelly tool. Next, an initial assembly of the second-generation data was performed using SPAdes, while the third-generation data were initially assembled with Canu (v2.1.1). Finally, the hybrid assembly was refined by integrating both datasets with Pilon (v1.23), which allowed for genome correction and the generation of high-quality, clean data in FASTA format.

### 2.5 Genetic analysis

Genome annotation was executed using nine databases, including the Comprehensive Antibiotic Resistance Database (CARD), Kyoto Encyclopedia of Genes and Genomes (KEGG), Clusters of Orthologous Groups (COG), Non-Redundant Protein Database (NR), Pathogen-Host Interaction (PHI), Swiss-Prot, Virulence Factor Database (VFDB). These databases were accessed and annotated through the Beijing Micro Future Pathogen Microbiological Information Analysis System platform. Circular genome maps were generated utilizing CGView online software (available at https://stothardresearch.ca/cgview/). Phylogenetic relationships and genomic homology were assessed using Average Nucleotide Identity (ANI) to compare the genomic sequences of the bacteria against reference databases, thus providing a measure of genetic relatedness (available at https://www.ezbiocloud.net/). Furthermore, DNA–DNA hybridization (DDH) was performed to quantitatively assess genomic similarity between the unknown strain and reference strains (accessible at https://ggdc.dsmz.de/).

### 2.6 Drug resistance phenotype

Antimicrobial susceptibility testing was performed using the CHN5GOVF customized panel (broth microdilution method, National Pathogen Identification Network, China) following the Clinical and Laboratory Standards Institute (CLSI) M100 guidelines (Humphries et al., 2021). The procedure included: Bacterial suspension preparation: Pure colonies of *Vagococcus fluvialis* were adjusted to a 0.5 McFarland standard (~1 × 10^8^ CFU/mL) in sterile saline and further diluted to a final concentration of 1 × 10^6^ CFU/mL. Inoculation and incubation: The bacterial suspension was dispensed into the panel wells and incubated aerobically at 35°C for 18–24 h. MIC determination: The minimum inhibitory concentration (MIC) was defined as the lowest drug concentration showing no visible growth. Susceptibility (S), intermediate (I), or resistance (R) were interpreted according to CLSI breakpoints. Quality control: *Escherichia coli* ATCC 25922 was included as a quality control strain in each run.

### 2.7 Motility assay

Motility was assessed by inoculating strains into semi-solid agar and performing a stab-inoculation method. After 24 h of incubation at 37°C, motility was determined by the diffusion of the inoculation line: non-diffusive growth indicated negative motility, while a blurred, diffused line indicated positive motility.

## 3 Results

### 3.1 Morphological characterization of strain 25C42

Following Gram staining, strain 25C42 exhibited a positive result, and the colony morphology was cocci (Supplementary Figure S1A). Motility assays indicated that strain 25C42 was non-motile, contrasting with the positive motility observed in the control strain, *Escherichia coli* ATCC25922 (Supplementary Figure S1B).

### 3.2 Phylogenetic relationships of 16S rRNA gene sequences

**Figure 1:**
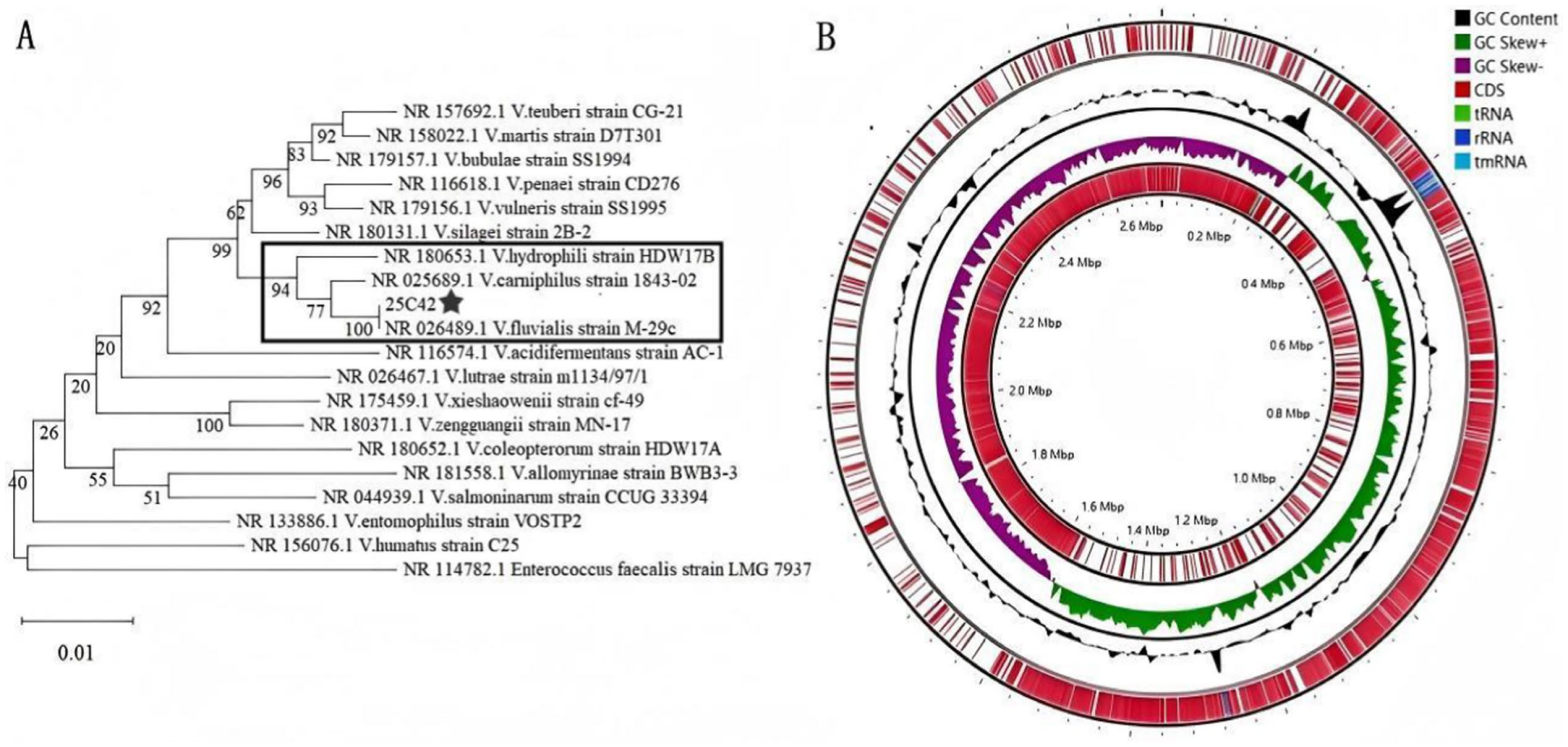
**(A)** Phylogenetic tree with 16S rRNA gene sequences of strains 25C42 and 18 other Vagococcus species. **(B)** Complete gene map of strain 25C42.

The identification of strain 25C42 through MALDI-TOF MS yielded a definitive classification as *V. fluvialis* with a high identification score of 8.285. Further confirmation was obtained via 16S rRNA gene sequencing, which showed 100.00% identity with *V. fluvialis* (NCBI accession number: NR_026489.1). The 16S rRNA gene sequence of strain 25C42 encompass 1,549 base pairs, we have uploaded the sequence to a public database.^1^ We downloaded the 16S rRNA sequences of all *Vagococcus* species similar to strain 25C42 from the NCBI database, totaling 18 species, the phylogenetic tree was rooted using *Enterococcus faecalis* strain LMG 7937. The NJ phylogenetic tree demonstrated that strain 25C42 formed a robust monophyletic cluster with *V. fluvialis* strain M-29c (NR_026489.1), supported by a maximum bootstrap value of 100% (Figure 1A). Additionally, strain 25C42 demonstrated a close phylogenetic relationship with *V. hydrophili* strain HDW17B (NR_180653.1) and *V. carnipilus* strain 1843-02 (NR_025689.1), both of which showed high bootstrap values. This topology effectively resolved the evolutionary relationships among Vagococcus species members. The observed phylogenetic patterns were consistently validated by three complementary reconstruction methods (ML, ME, and MP), as illustrated in Supplementary Figures S2–S4.

### 3.3 Complete gene map of strain 25C42

By integrating both second and third-generation WGS data for assembly and gene prediction, we achieved a genome assembly integrity of 99.92%, maintaining a splicing quality of 100% and a reduced contamination level of 1.38%. The annotated genome comprises 77 tRNA genes and 7 copies each of the 23S, 16S, and 5S rRNA genes. The total sequence length is 2,720,341 bp, characterized by a GC content of 32.57%. The assembly yielded a single contig with no gaps, exhibiting N50 and N75 values both at 2,720,341 bp. We’ve uploaded the sequence to a public database.^2^ Based on the criteria established by Bowers et al. (2017) and Duan et al. (2020), this genome sequence was classified as complete and of high quality. The complete gene map features a circular representation of the coding regions, color-coded according to functional categories, and includes information on non-coding RNAs (tmRNA, tRNA, rRNA), GC content, and coding DNA sequences (CDS) (Figure 1B).

### 3.4 Functional gene analysis

The complete gene sequence of strain 25C42 underwent annotation across six databases, yielding 2,268 genes in the COG database, 2,023 in KEGG, 133 in PHI, 2,610 in NR, 119 in VFDB, 65 in CARD.

**Figure 2:**
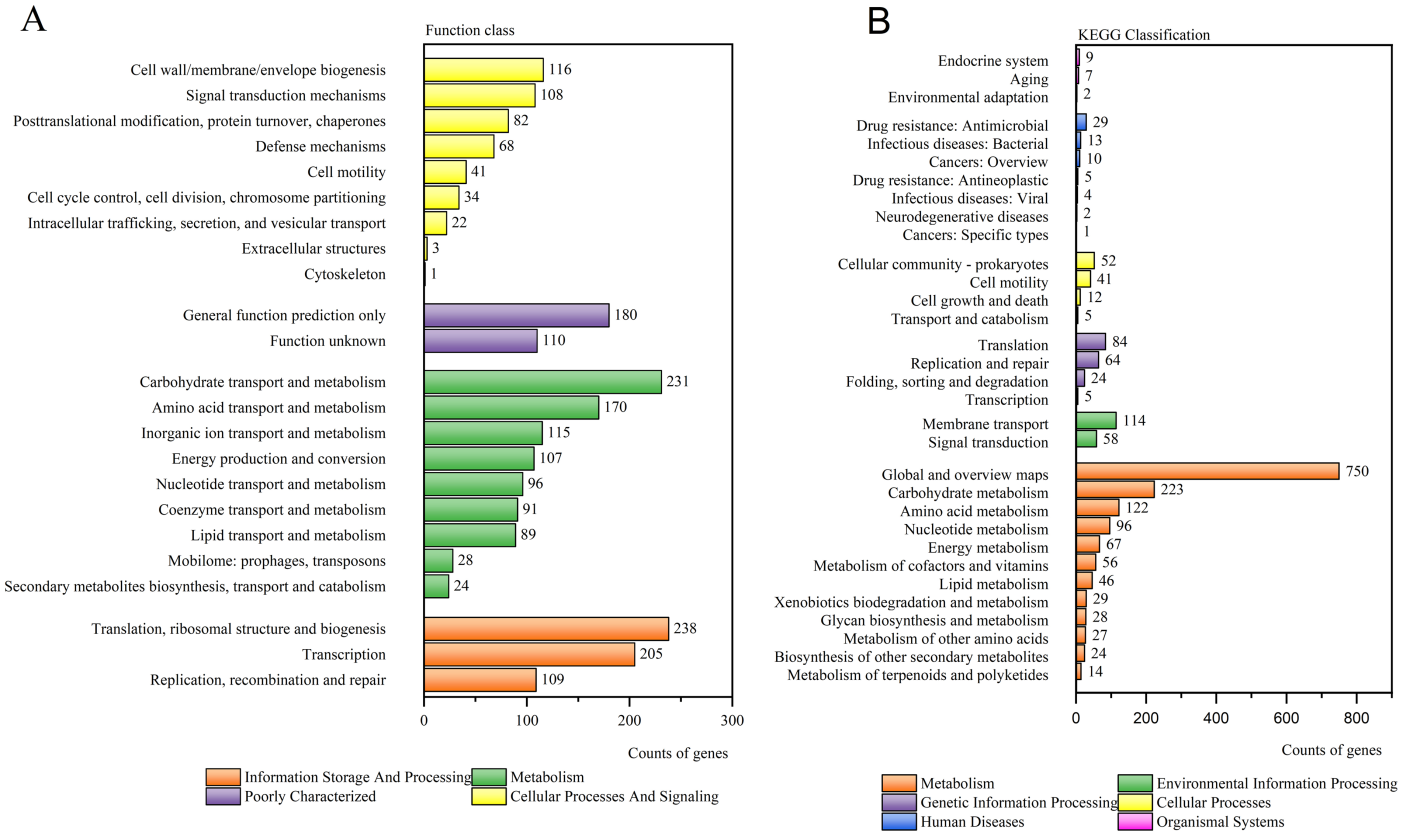
**(A)** COG analysis of strain 25C42. **(B)** KEGG analysis of strain 25C42.

COG analysis classified the 2,268 annotated genes into four primary categories and 23 functional groups. The major categories included Cellular Processes and Signaling, Information Storage and Processing, Metabolism, and Poorly Characterized functions. Notably, the most prevalent annotations were found in “Translation, ribosomal structure and biogenesis” (238 genes), “Carbohydrate transport and metabolism” (231 genes), and “Transcription” (205 genes). Other significant functional annotations encompassed cell wall/membrane/envelope biogenesis, inorganic ion transport and metabolism, and defense mechanisms (Figure 2A).

A total of 2,023 orthologous protein-coding genes were mapped to 32 KEGG metabolic pathways, categorized into Cellular Processes, Environmental Information Processing, Genetic Information Processing, Human Diseases, Metabolism, and Organismal Systems. The predominant pathways identified were related to Metabolism, including “Global and overview maps” (750 genes), “Carbohydrate metabolism” (223 genes), and “Amino acid metabolism” (122 genes). Additionally, Environmental Information Processing pathways, such as “Membrane transport” (114 genes), and pathways associated with Human Diseases, including “Drug resistance: Antimicrobial” (29 genes) and “Infectious diseases: Bacterial” (13 genes), were also noted (Figure 2B).

**Figure 3:**
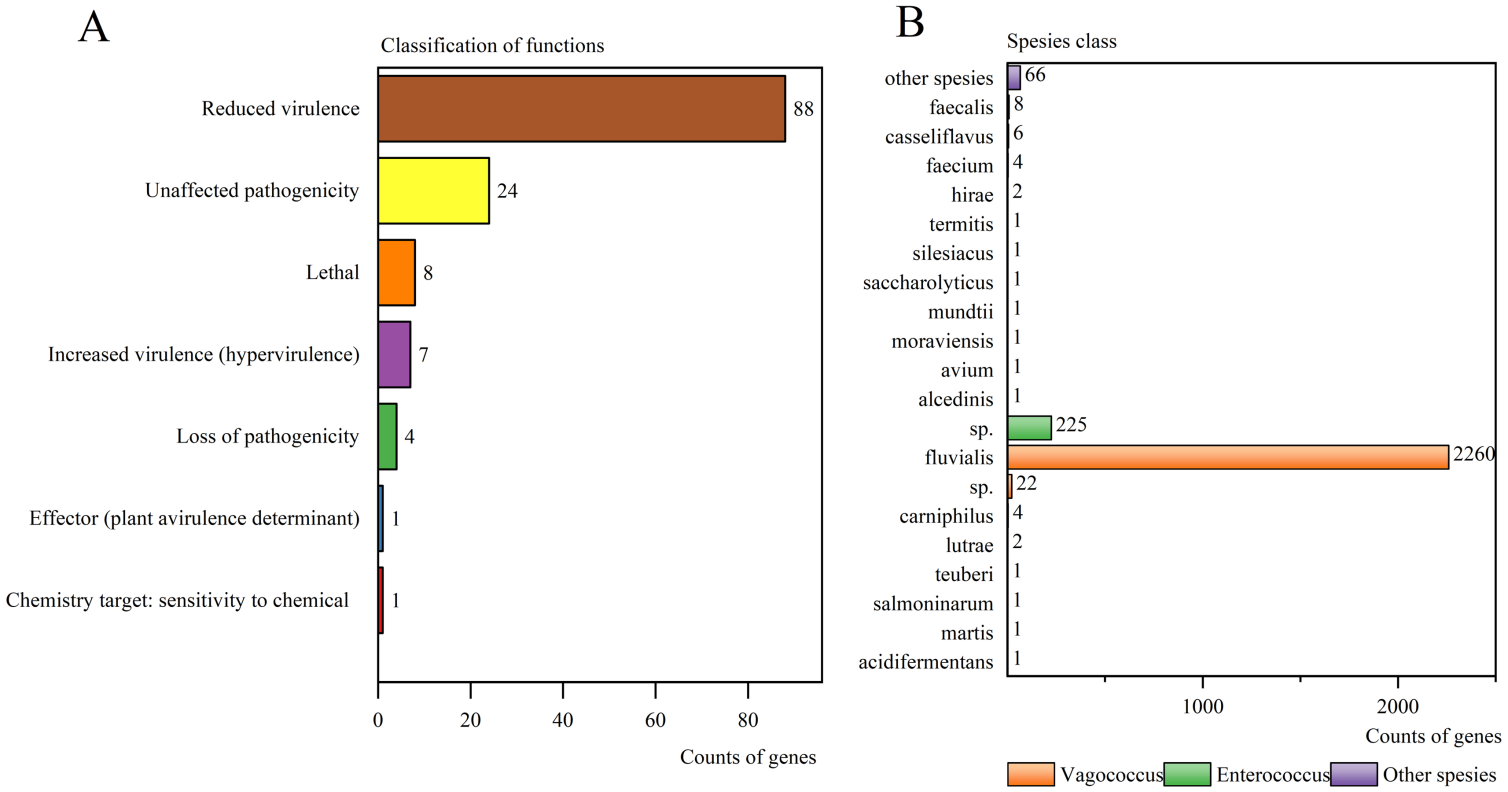
**(A)** Types distribution of PHI genes of strain 25C42. **(B)** NR analysis of strain 25C42.

Pathogen-host interaction-related genes were examined with 133 genes annotated in the PHI database. These were categorized into 7 groups, with “Reduced virulence” (88 genes) being the most prominent, followed by “Unaffected pathogenicity” (24 genes), “Lethal” (8 genes), “Increased virulence (hypervirulence)” (7 genes), “Loss of pathogenicity” (4 genes), “Effector (plant avirulence determinant)” (1 genes), and “Chemistry target: sensitivity to chemical” (1 gene) (Figure 3A).

In the NR database, the gene sequence of strain 25C42 was translated into amino acid sequences, revealing 2,610 annotated genes. Notably, *V. fluvialis* was the most frequently annotated, comprising 86.59% of the annotations, followed by *Enterococcus* sp. at 8.62%, and *V.* sp. accounts for 0.84% (Figure 3B).

**Figure 4:**
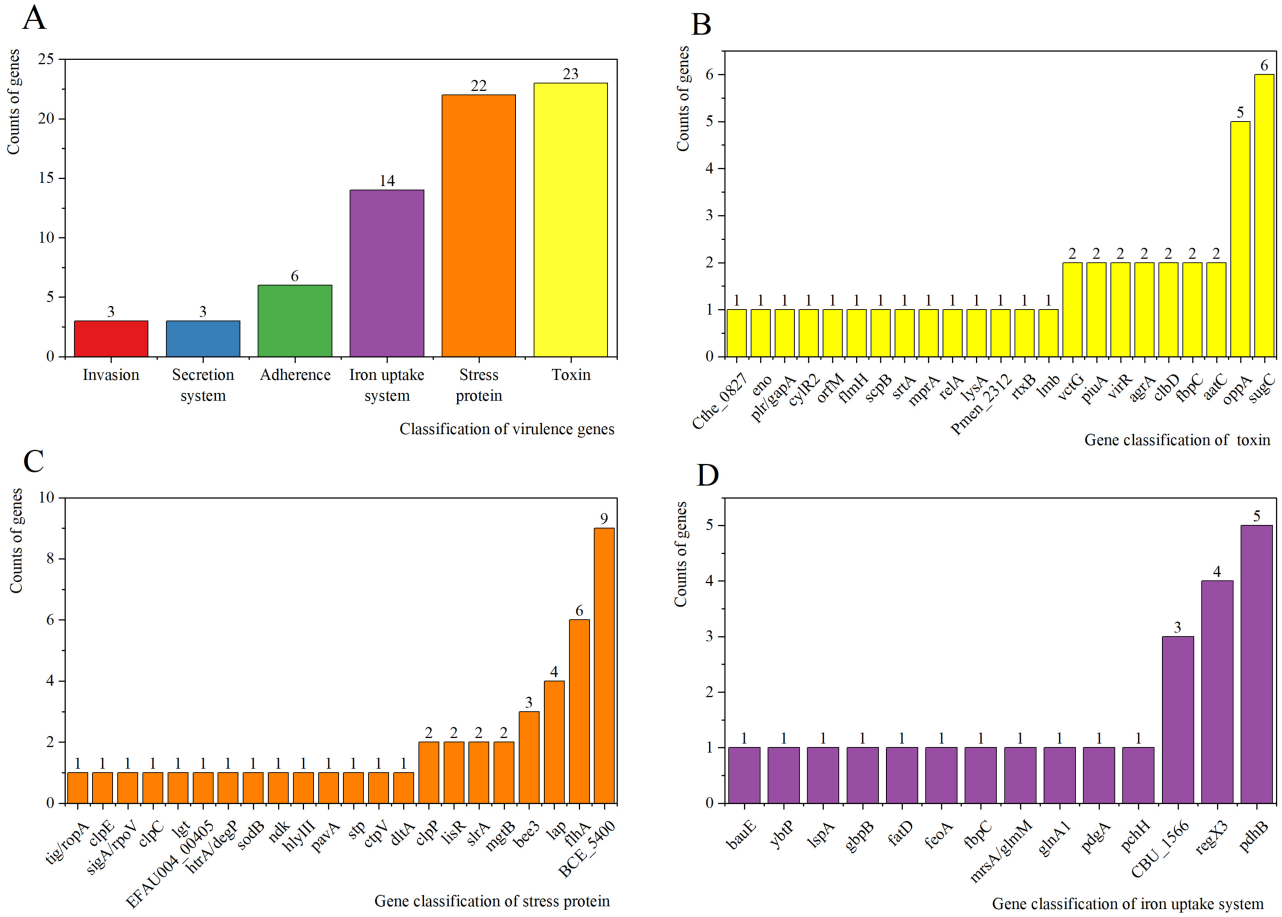
**(A)** VFDB analysis of strain 25C42. **(B)** Results of virulence gene distribution in toxin. **(C)** Results of virulence gene distribution in stress protein. **(D)** Results of virulence gene distribution in iron uptake system.

Virulence factors were classified into 6 functional categories: Invasion (3 genes), Secretion system (3 genes), Adherence (6 genes), Iron uptake system (14 genes), Stress protein (22 genes), and Toxin (23genes). DIAMOND analysis against the VFDB identified 119 putative virulence genes in strain 25C42, positive results were accepted with at least 40% identity (Figure 4A). There were 13 virulence genes with more than 60% identity (Supplementary Table 1). With the highest number of annotated genes associated with carbohydrate ABC transporter ATP-binding (sugC) and peptide/nickel transport system substrate-binding protein (oppA) in Toxin (Figure 4B). With the highest number of annotated genes associated with flagellar biosynthesis protein FlhA (flhA), capsular exopolysaccharide family protein (BCE_5400) and putative alcohol-acetaldehyde dehydrogenase (lap) in Stress protein (Figure 4C). With the highest number of annotated genes associated with DNA-binding response regulator RegX3 (regX3), e1-beta chain (pdhB) and Coxiella Dot/Icm type IVB secretion system translocated effector (CBU_1566) in Iron uptake system (Figure 4D). In addition, Secretion system includes bactoprenol glucosyl transferase (gtrB), capsular polysaccharide biosynthesis protein Cap5H (cap5H), Secretion System Chemotaxi-specific methylesterase (cheB). Invasion contains UDP-glucose pyrophosphorylase (hasC), chemotaxis protein CheA (cheA), type IV pilus response regulator PilG (pilG). Adherence includes Elongation factor Tu (tuf), chaperonin GroEL (groEL), autolysin (lytA), endocarditis specific antigen (efaA), molecular chaperone DnaK (CT396), putative lipoate protein ligase A (lplA1).

**Figure 5:**
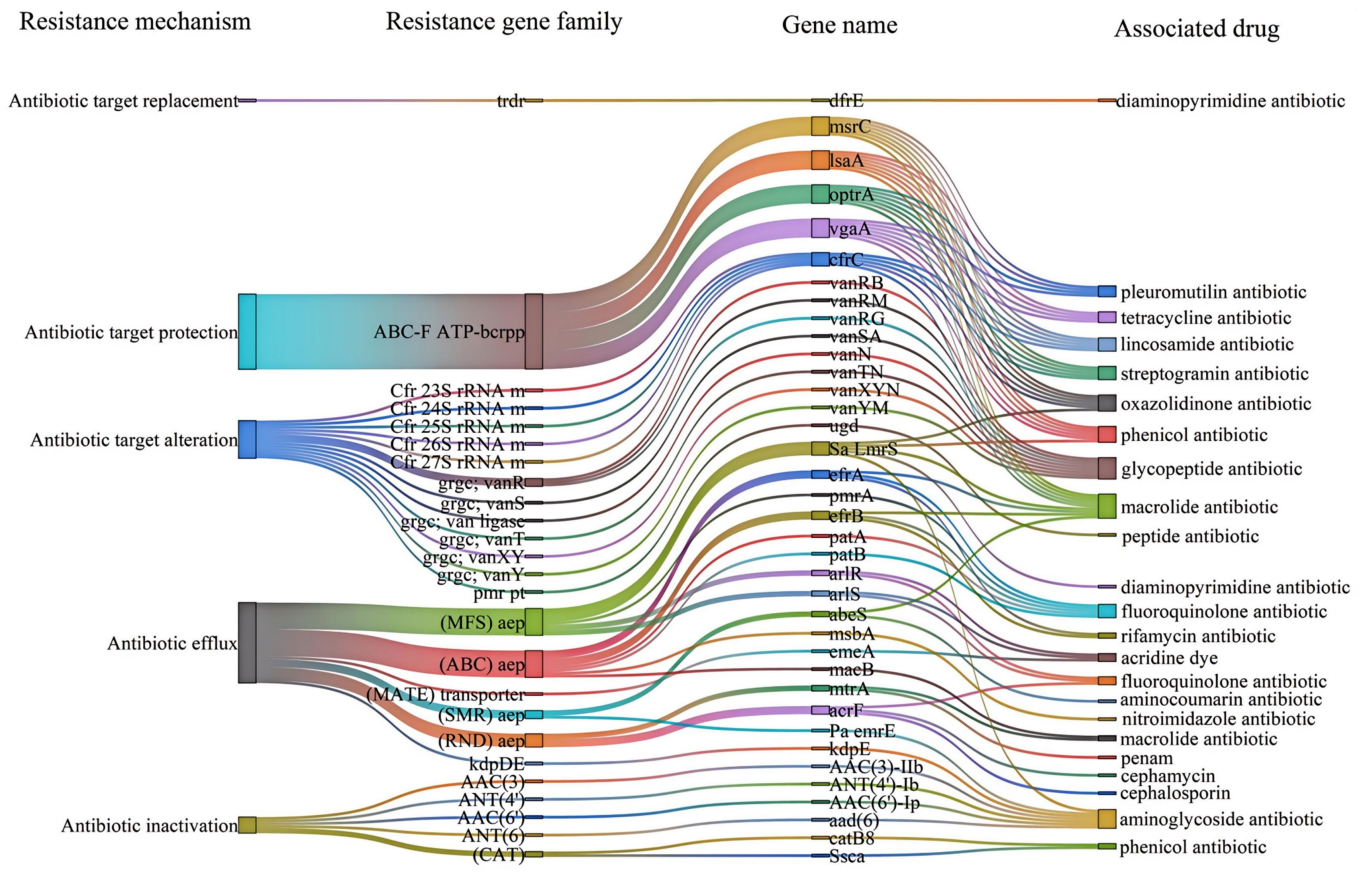
Drug-resistant genes of strain 25C42 annotated in the CARD database (identity > 40%). ABC-F, ABC-binding cassette ribosomal protection protein; ABC, ATP-binding cassette; Cfr 23S rRNA m, Cfr 23S ribosomal RNA methyltransferase; CAT, chloramphenicol acetyltransferase; grgc, glycopeptide resistance gene cluster; aep, antibiotic efflux pump; MFS, major facilitator superfamily; MATE, multidrug and toxic compound extrusion transporter; pmr pt., pmr phosphoethanolamine transferase; RND, resistance-nodulation-cell division; SMR, small multidrug resistance; trdr, trimethoprim-resistant dihydrofolate reductase (dfr); Pa emrE, *Pseudomonas aeruginosa* emrE; Sa LmrS, *Staphylococcus aureus* LmrS; Ssca, *Streptococcus suis* chloramphenicol acetyltransferase.

The CARD database annotated 65 antimicrobial resistance genes from strain 25C42 (identity >40%) (Supplementary Table 2). Identifying five primary resistance mechanisms: Antibiotic efflux, Antibiotic inactivation, Antibiotic target alteration, Antibiotic target protection, and Antibiotic target replacement. The resistance gene families included 21 distinct types, notably the ABC-F ATP-binding cassette ribosomal protection protein, major facilitator superfamily (MFS) antibiotic efflux pump, and glycopeptide resistance gene cluster. Predictions of drug resistance indicated the potential for resistance to a wide range of antibiotics, including acridine dyes, fluoroquinolone antibiotics, and others (Figure 5).

### 3.5 Whole genome similarity comparisons

**Figure 6:**
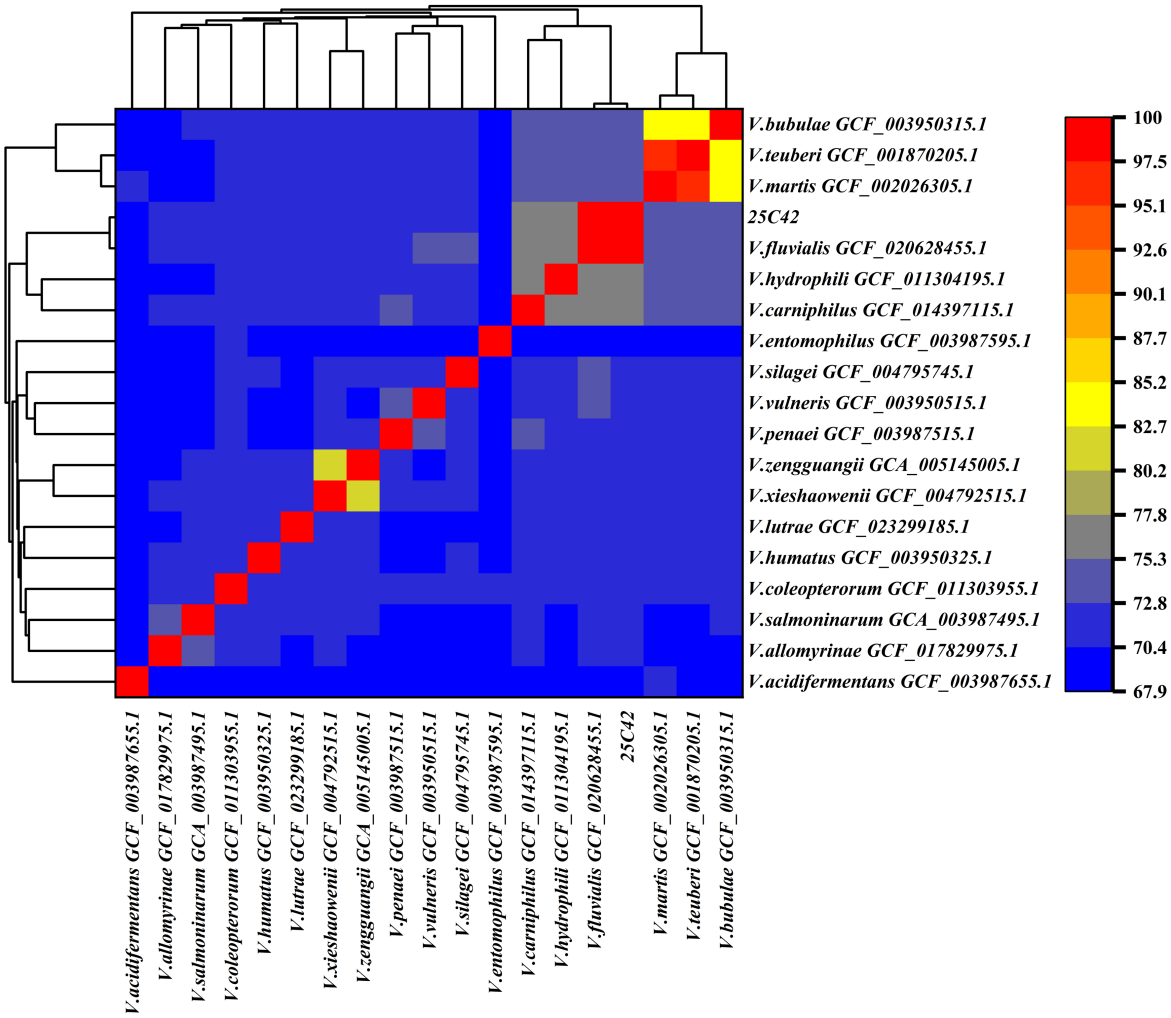
Heat map of ANI between strain 25C42 and 18 phylogentically close *Vagococcus* species.

For comparative genomic analysis, strain 25C42 was selected for ANI and DDH assessment against 18 other *Vagococcus* strains with complete genome sequences and closely related phylogenetic backgrounds. The ANI value between strain 25C42 and *V. fluvialis* (GCF_020628455.1) was determined to be 98.57%, exceeding the 95% threshold indicative of significant genomic similarity. Furthermore, strain 25C42 clustered closely with *V. hydrophili* (GCF_011304195.1) and *V. carnipilus* (GCF_014397115.1). Additionally, the ANI value between *V. Martis* (GCF_002026305.1) and *V. teuberi* (GCF_001870205.1) was calculated at 95.77% (Figure 6). For the DDH analysis, strain 25C42 was compared with 18 closely related *Vagococcus* strains using all three default calculation formulas. The highest DDH value obtained was 88.6% (DDH ≥ 70%), recorded in comparison with *V. fluvialis* (GCF_020628455.1), yielding a probability of 95.32% (Supplementary Table 3).

### 3.6 Antimicrobial resistance profile

The strain exhibited resistance to clindamycin, tetracycline, rifampin, cefoxitin, and levofloxacin (Table 1). Additionally, penicillin resistance may be related to the activity of natural *β*-lactamase (Rodriguez Jimenez et al., 2022). The strain was sensitive to erythromycin, while resistant to clindamycin, which could suggest erm-mediated inducible resistance, requiring confirmation through a D-test (Hollenbeck and Rice, 2012).

**Table 1:** Antimicrobial susceptibility profile of strain 25C42.

Class	Antibiotic	Abbreviation	MIC (μg/mL)	Interpretation
β-Lactams	Penicillin	PEN	0.5	R^*^
	Cefoxitin	CFX	>8	R
Macrolides/Lincosamides	Clindamycin	CLI	>8	R
	Erythromycin	ERY	≤0.5	S
	Erythromycin/Clindamycin	ERY/CLI	≤4/0.5	S/R^†^
Tetracyclines	Tetracycline	TET	>16	R
Rifamycins	Rifampicin	RIF	>4	R
Fluoroquinolones	Levofloxacin	LEV	4	R
Glycopeptides	Vancomycin	VAN	1	S
	Teicoplanin	TEC	≤0.5	S
Oxazolidinones	Linezolid	LZD	≤1	S
Lipopeptides	Daptomycin	DAP	0.5	S
Aminoglycosides	Gentamicin	GEN	0.5	S
Folate pathway inhibitors	Cotrimoxazole	SXT	≤0.25	S
Nitrofurans	Nitrofurantoin	NIT	≤16	S
Other	Oxacillin	OXC	≤0.12	S

“R” indicates resistant; “S” indicates sensitive; R^*^, Penicillin resistance may be related to natural beta-lactamase activity; S/R^†^, Erythromycin sensitive but clindamycin resistant suggests potential induced resistance (D test required).

## 4 Discussion

The emergence of *V. fluvialis* as a potential human pathogen poses significant challenges within microbiology and clinical medicine. Historically regarded as a less prominent member of the *Enterococcaceae* family, recent findings indicate its isolation from a diverse range of hosts, including humans, bats, and livestock. This suggests a broader ecological niche than previously recognized. Our study reports the first isolation of the more resistant *V. fluvialis* in wild *Niviventer*, contributing to the growing understanding of this bacterium’s presence in mammals. By investigating the resistance and pathogenicity of strains isolated from novel hosts, we aim to elucidate their potential public health implications and provide essential data for future environmental monitoring and the development of antimicrobial therapeutics. These findings not only enhance our comprehension of the transmission and adaptation mechanisms of *V. fluvialis* across different ecosystems but also offer a new avenue for research in the prevention and control of related infections.

The identification of strain 25C42 as *V. fluvialis* was substantiated through comprehensive analyses employing both MALDI-TOF mass spectrometry and 16S rRNA gene sequencing, achieving a 100% identity match with established *V. fluvialis* sequences (NCBI number: NR_026489.1). This underscores the reliability of these methodologies for accurate identification, aligning with findings from prior studies (Chen et al., 2024; Kitano et al., 2024).

Our sequencing efforts yielded a high-quality genome assembly with an integrity of 99.92% and a contamination level of 1.38%, demonstrating the efficacy of integrating second-and third-generation sequencing technologies for thorough genomic analysis. The assembled genome spans a total length of 2,720,341 bp with a GC content of 32.57%, further affirming its classification within the *Vagococcus* genus, as similar GC content ranges have been reported in closely related species (Lewis and Jorgensen, 2005).

Gene annotation via Prokka unveiled a diverse array of functional genes categorized into multiple biological processes. Notably, the predominant functions identified encompassed translation, ribosomal structure, carbohydrate transport, and amino acid metabolism. These results are consistent with the observations of Rodriguez Jimenez et al. (2022), who highlighted metabolic versatility as a defining trait of *Vagococcus* species, enabling adaptation to fluctuating environmental conditions. The comprehensive mapping of 2,268 genes to COG functional categories illustrates a complex regulatory and metabolic network, which is essential for the organism’s survival and pathogenic potential.

The pathogenicity potential of strain 25C42 is further accentuated by the identification of 133 pathogen-host interaction-related genes, predominantly linked to reduced virulence. This observation suggests that many environmental *Vagococcus* species harbor genes that may modulate virulence without necessarily conferring hypervirulence. Furthermore, the identification of virulence factors, including secretion systems and iron uptake mechanisms, corroborates the findings of Jimenez Ana et al., highlighting the significance of these factors in the pathogenicity of *Vagococcus* species (Lewis and Jorgensen, 2005).

This study is the first to report the antibiotic susceptibility profile of *Vagococcus fluvialis* to 16 antibiotics in the CHN5GOVF panel. The antimicrobial resistance profile of strain 25C42 is particularly concerning. Resistance to clindamycin, tetracycline, rifampicin, and cefoxitin reflects an alarming trend in *V. fluvialis*, as noted in studies where strains isolated from infected patients exhibited poor antibacterial efficacy against clindamycin, oxacillin, and sulfamethoxazole/trimethoprim (Ting et al., 2019). Additionally, *V. fluvialis* isolated from bats demonstrated potential resistance to macrolides (Qin et al., 2021). The results indicated multi-drug resistance, with the strain showing resistance to clindamycin, tetracycline, rifampin, cefoxitin, and levofloxacin, suggesting the presence of multiple resistance genes, such as erm, tet, and gyrA mutations (RDarby et al., 2023). Susceptible antibiotics included glycopeptides (vancomycin, teicoplanin), aminoglycosides (gentamicin), and linezolid (Arias and Murray, 2012), offering alternative treatment options. The minimum inhibitory concentration (MIC) of penicillin was 0.5 μg/mL (determined resistant by CLSI), which may reflect *β*-lactamase activity or mutations in penicillin-binding proteins (PBPs) (Queenan and Bush, 2007), and should be further verified by molecular testing (e.g., blaZ gene detection). The sensitivity to erythromycin and resistance to clindamycin may indicate erm-mediated inducible resistance, which needs to be confirmed by a D-test (Hollenbeck and Rice, 2012). Future studies are warranted to determine whether different sources of *V. fluvialis* exhibit distinct resistance patterns or resistance genes.

Genomic analysis revealed 65 antibacterial resistance genes in strain 25C42, many of which correlate with its phenotypic resistance. For example, lsa (A) and msrC likely mediate clindamycin resistance through ribosomal protection and efflux, while tet (M) and tet (L) synergistically confer tetracycline resistance. Similarly, fluoroquinolone resistance (levofloxacin) aligns with efflux pumps (pmrA, qacA) and potential gyrA mutations. However, some discrepancies exist: rifampicin resistance (MIC > 4 μg/mL) may stem from rpoB mutations not captured by CARD, and cefoxitin resistance could involve unannotated β-lactamases or PBPs. Notably, erythromycin sensitivity despite erm (B) presence suggests inducible resistance, requiring phenotypic validation via D-test. These findings underscore the importance of integrating genomic predictions with phenotypic assays to fully resolve resistance mechanisms, particularly for emerging pathogens like *V. fluvialis*.

Identified mechanisms of resistance, such as antibiotic efflux and target alteration, mirror patterns observed in other pathogenic bacteria, indicating shared evolutionary pathways (Jiang et al., 2018; Guitor and Wright, 2018). This finding emphasizes the critical need for ongoing surveillance of antimicrobial resistance patterns in *V. fluvialis* to mitigate the public health risks associated with these emerging pathogens.

Phylogenetic analyses revealed that strain 25C42 is closely related to *V. fluvialis* strain M-29c, with a high Average Nucleotide Identity (ANI) value of 98.57% and robust DNA–DNA Hybridization (DDH) values further supporting its classification. Collectively, these findings illuminate the intricate interplay between environmental adaptation, pathogenicity, and antimicrobial resistance in *V. fluvialis*, underscoring the necessity for further investigations to unravel the implications of these traits in clinical and environmental contexts.

The limitations of this study include the lack of detection of resistance genes and the absence of animal model validation.

## 5 Conclusion

Our findings highlight the emergent role of *V. fluvialis* as a potential human pathogen, revealing its resistance mechanisms and pathogenicity in diverse hosts. The high genomic integrity and extensive functional gene annotation underscore its metabolic versatility. Notably, strain 25C42 exhibits significant antimicrobial resistance, necessitating ongoing surveillance and research to understand its implications for public health and environmental monitoring, as well as strategies for effective therapeutic intervention.

## Data Availability

The datasets generated and/or analyzed during the current study are available in the Genome Sequence Archive (GSA) repository, 16S rRNA gene sequence: https://bigd.big.ac.cn/gsa/browse/CRA021120 (accession number: CRA021120); whole genome sequences: https://bigd.big.ac.cn/gsa/browse/CRA021105 (accession number: CRA021105).

## Footnotes

^1^ https://bigd.big.ac.cn/gsa/browse/CRA021120

^2^ https://bigd.big.ac.cn/gsa/browse/CRA021105

## Supplementary material

Supplementary Table 1 VFDB.

Supplementary Table 2 CARD.

Supplementary Table 3 DDH values between 25C42 and 18 phylogenetically close Vagococcus species.

SUPPLEMENTARY FIGURE 1 **(A)** Morphology of strain 25C42 under microscope (100x oil lens). **(B)** Motility assays observation (*Escherichia coli* ATCC25922 was the control group).

SUPPLEMENTARY FIGURE 2 Phylogenetic tree(ML) with 16S rRNA gene sequences of strains 25C42 and 18 other *Vagococcus* species.

SUPPLEMENTARY FIGURE 3 Phylogenetic tree(ME) with 16S rRNA gene sequences of strains 25C42 and 18 other *Vagococcus* species.

SUPPLEMENTARY FIGURE 4 Phylogenetic tree(MP) with 16S rRNA gene sequences of strains 25C42 and 18 other *Vagococcus* species.
