# Supplementary figures and images for: Whole-genome analysis and antimicrobial resistance phenotype of Vagococcus fluvialis isolated from wild Niviventer
Source: Front Microbiol. 2025 Apr 16;16:1546744. doi: 10.3389/fmicb.2025.1546744 (PMC12042758; doi:10.3389/fmicb.2025.1546744)

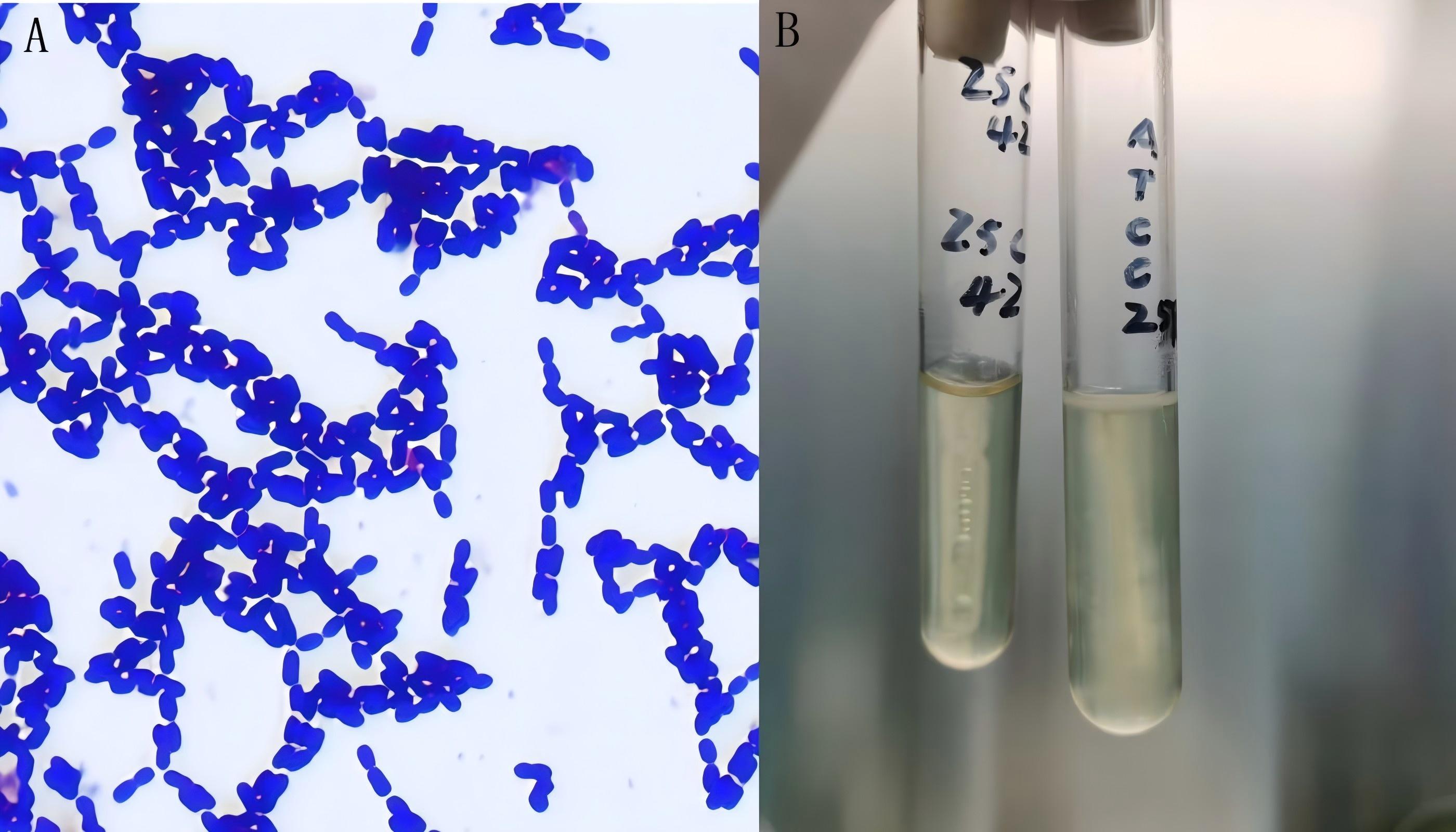

Supplement: SUPPLEMENTARY FIGURE 1 — (A) Morphology of strain 25C42 under microscope (100x oil lens). (B) Motility assays observation (Escherichia coli ATCC25922 was the control group). [file Image_1.jpg]

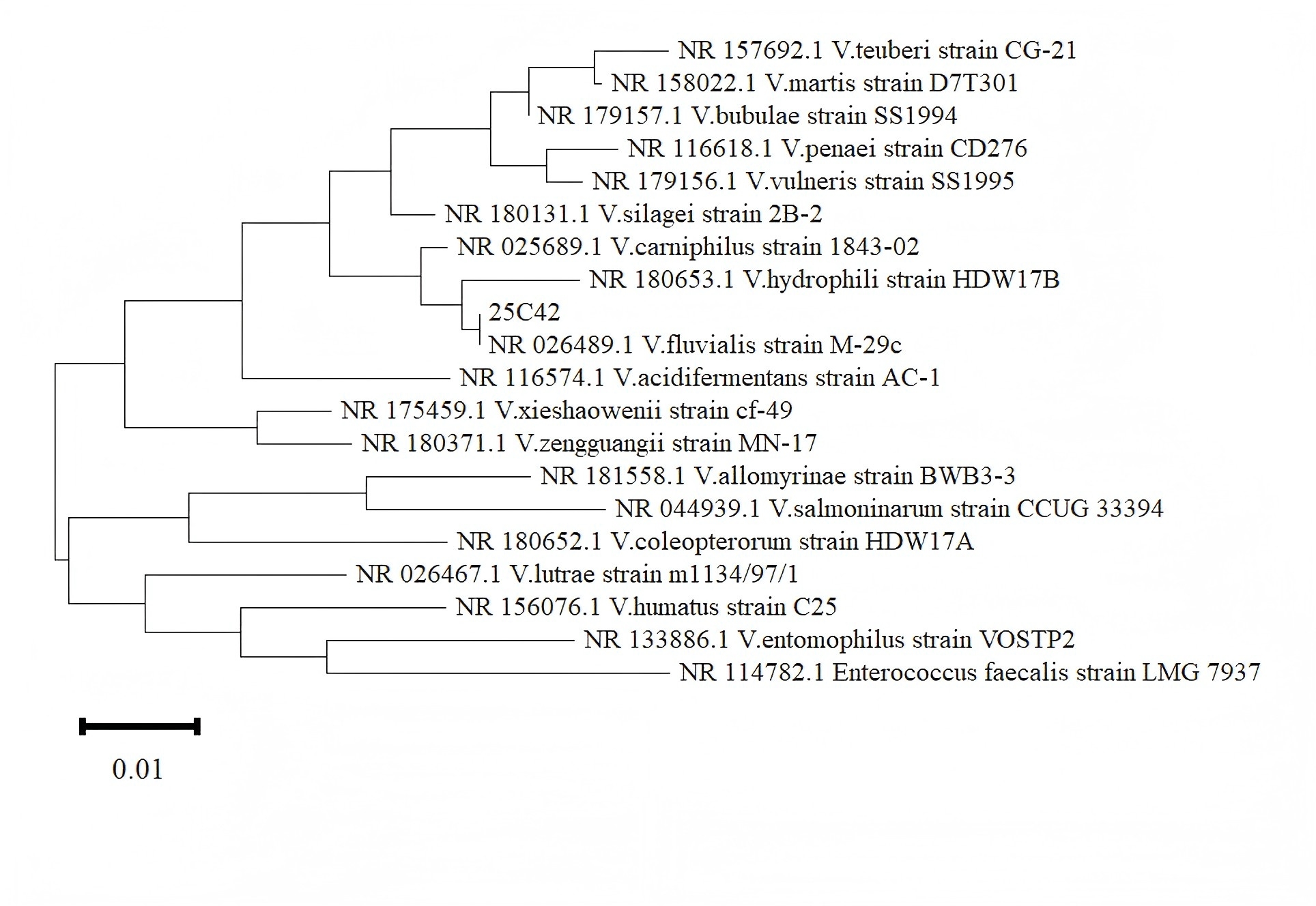

Supplement: SUPPLEMENTARY FIGURE 2 — Phylogenetic tree(ML) with 16S rRNA gene sequences of strains 25C42 and 18 other Vagococcus species. [file Image_2.jpg]

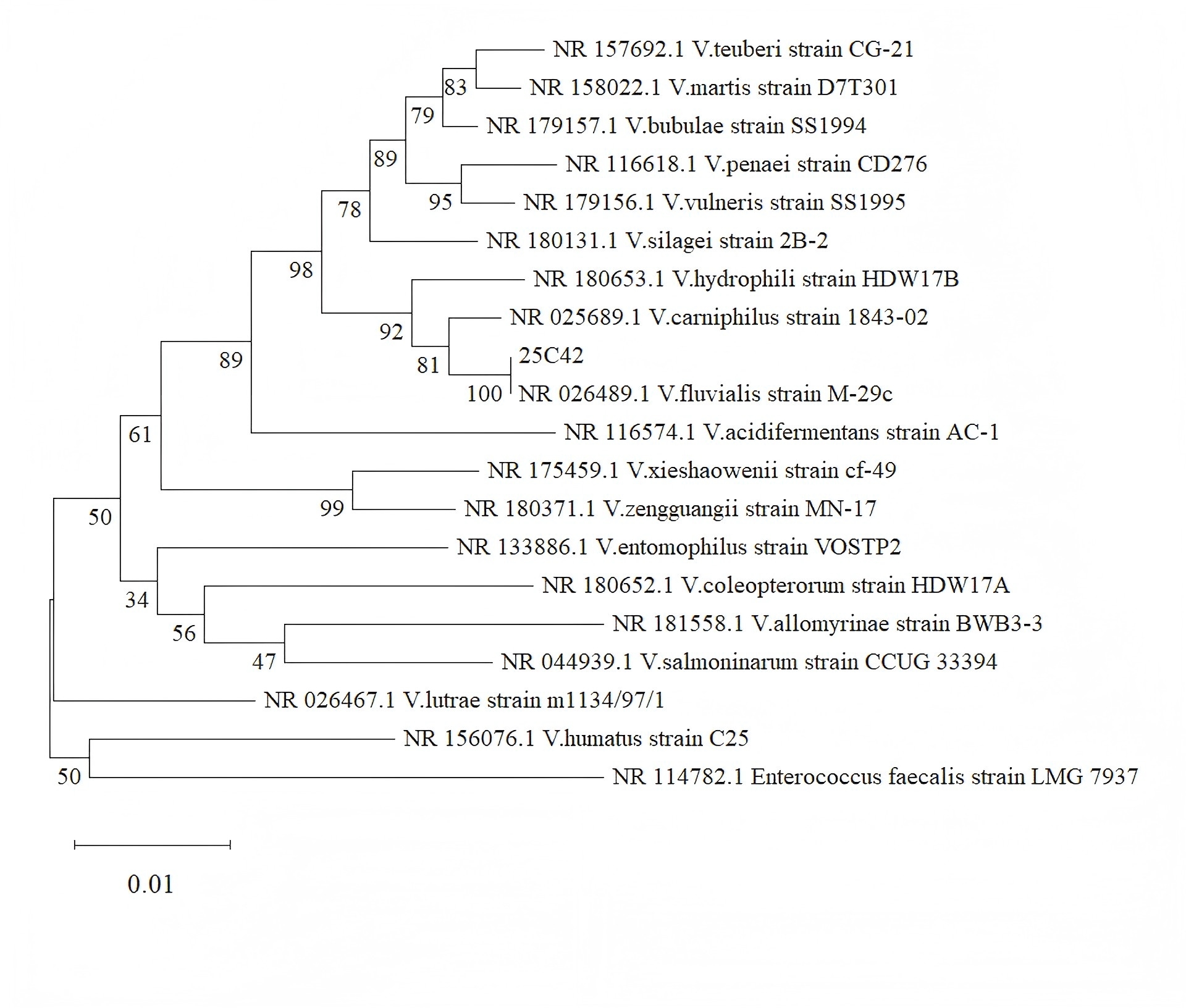

Supplement: SUPPLEMENTARY FIGURE 3 — Phylogenetic tree(ME) with 16S rRNA gene sequences of strains 25C42 and 18 other Vagococcus species. [file Image_3.jpg]

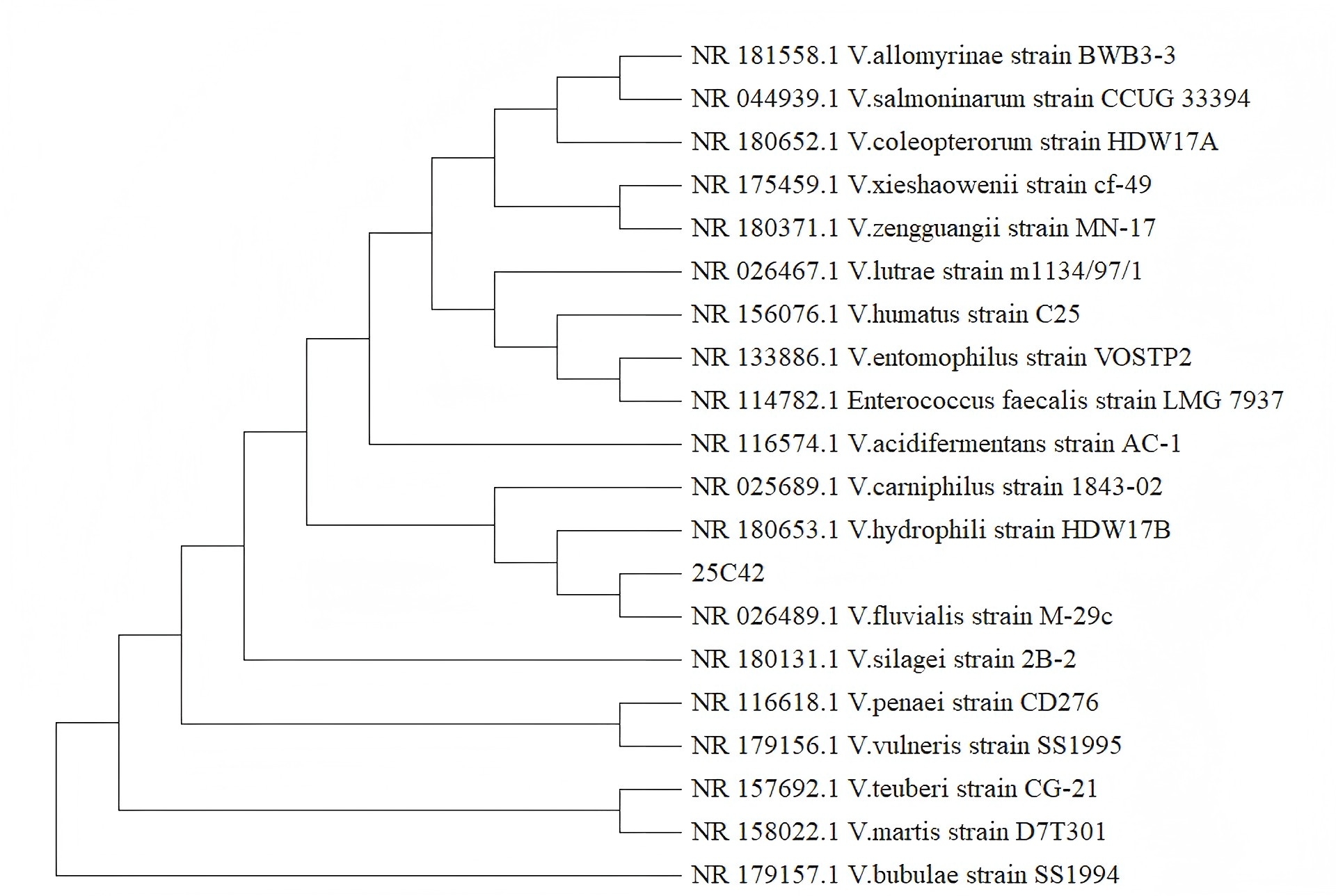

Supplement: SUPPLEMENTARY FIGURE 4 — Phylogenetic tree(MP) with 16S rRNA gene sequences of strains 25C42 and 18 other Vagococcus species. [file Image_4.jpg]
